# Monoamine oxidase-B (MAO-B) inhibitors: implications for disease-modification in Parkinson’s disease

**DOI:** 10.1186/2047-9158-2-19

**Published:** 2013-09-08

**Authors:** Kay Cheong Teo, Shu-Leong Ho

**Affiliations:** 1 Division of Neurology, Department of Medicine, University of Hong Kong, Hong Kong, China; 2 Research Centre of Heart, Brain, Hormone and Healthy Aging (HBHA), University of Hong Kong, Hong Kong, China

**Keywords:** Parkinson's disease, Monoamine oxidase-B inhibitors, Disease-modification, Neuroprotection, Selegiline, Rasagiline

## Abstract

There is a substantial amount of evidence from experimental parkinsonian models to show the neuroprotective effects of monoamine oxidase-B (MAOB) inhibitors. They have been studied for their potential disease-modifying effects in Parkinson’s disease (PD) for over 20 years in various clinical trials. This review provides a summary of the clinical trials and discusses the implications of their results in the context of disease-modification in PD. Earlier clinical trials on selegiline were confounded by symptomatic effects of this drug. Later clinical trials on rasagiline using delayed-start design provide newer insights in disease-modification in PD but success in achieving the aims of this strategy remain elusive due to obstacles, some of which may be insurmountable.

## Introduction

**Figure 1:**
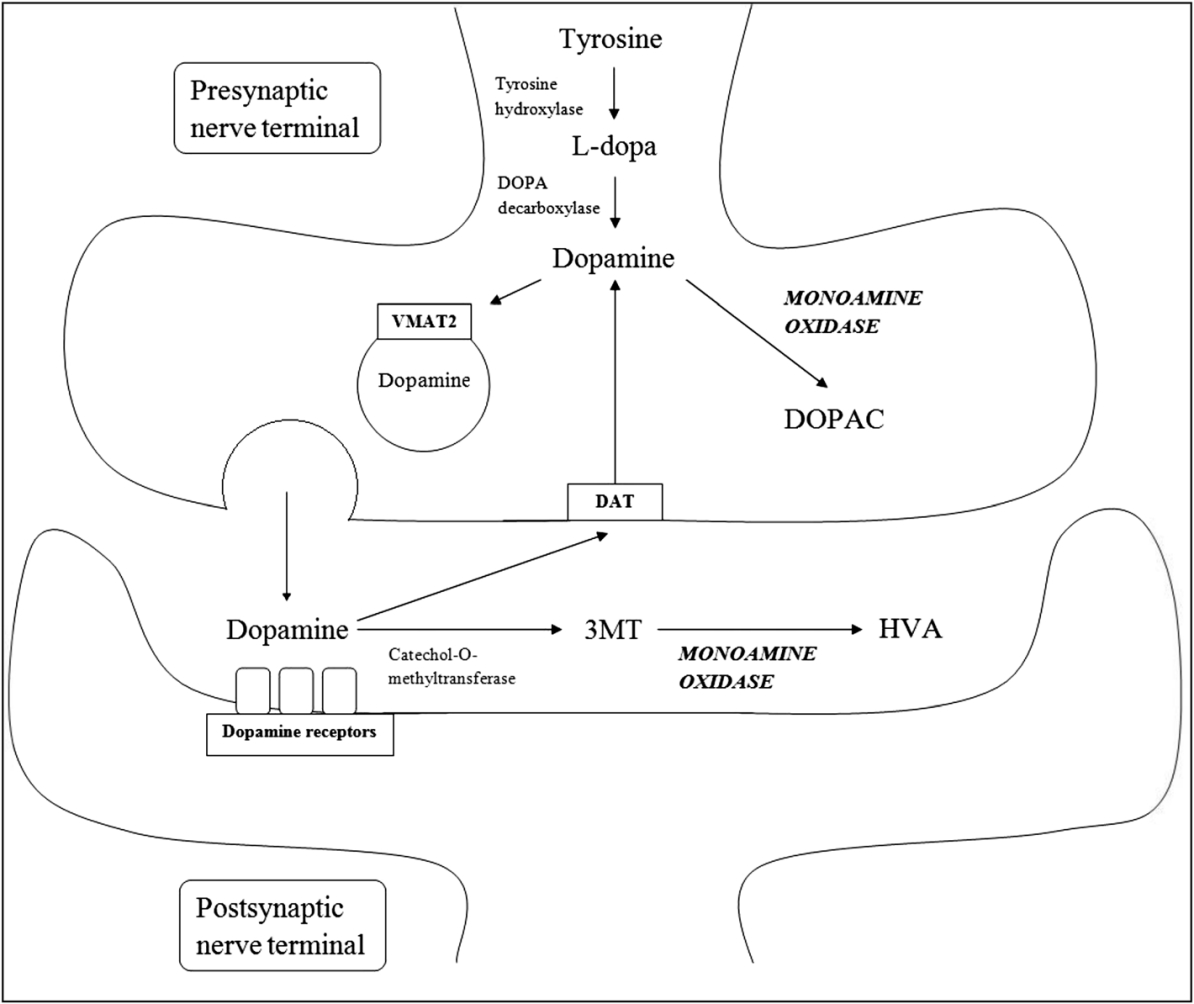
**Dopamine metabolism and the action of monoamine oxidase.** Abbreviations: VMAT2, vesicular monoamine transporter 2; DAT, dopamine active transporter; DOPAC, 3,4-dihydroxyphenylacetic acid; 3MT, 3-methoxytyramine; HVA, homovanillic acid.

For the purposes of this review, the term “disease-modification” refers to a broad definition of therapies which can slow or favorably modify the progressive degenerative processes in dopaminergic and non-dopaminergic neurons associated with Parkinson’s disease (PD). In this context, the term “neuroprotection” has been used almost synonymously with “disease-modification”. Monoamine oxidase-B (MAOB) is an enzyme that is involved in dopamine metabolism (Figure 1). MAOB inhibitors, namely selegiline and rasagiline, have been studied extensively for disease-modification in PD. This review will cover the rationale of this therapeutic strategy, clinical trials on MAOB inhibitors, the debates and caveats of the conclusions of these studies, and discuss the issues in deciding whether newer monoamine oxidase-B inhibitors such as rasagiline should be prescribed in the context of disease-modification in PD.

The incidence of PD increases with age, and is associated with significant disability. Its prevalence in patients over 50 years of age is projected to double in the ten most populous nations over the next two decades [1]. Classical motor features of PD such as bradykinesia, rigidity and tremor develop when about 50% of dopaminergic nigrostriatal neurons and about 80% striatal dopamine production are lost [2]. However, non-dopaminergic neurons pathways also undergo degeneration in PD, including cholinergic, noradrenergic, serotonergic and GABAergic pathways, resulting in a variety of non-motor features such as dementia, psychosis, depression and apathy. Unlike motor features which respond well to dopaminergic therapies such as levodopa and dopamine agonists, non-motor features which have either no or little response to dopaminergic therapies, can be more disabling to the patient and have a far greater impact on the quality of life. As yet, there is no conclusive proof that current therapy can delay, halt or reverse the neuronal degeneration in PD. Existing drug treatment is associated with a gradual loss of efficacy and long term side effects. Although stereotactic deep brain stimulation can relieve some motor symptoms, other motor and non-dopaminergic features are not relieved. As symptom-onset in most PD patients occur in their early 60s, even if partial disease-modification can be achieved, the progression to severe disability may be delayed past the patients’ natural life expectancy, so as to provide them with a satisfactory quality of life.

### Selegiline

Selegiline (N-Propargyl-methamphetamine) is a selective, irreversible MAOB inhibitor at therapeutic dose of 10 mg/day, but loses its selectivity at greater dosage [3]. Hence, selegiline at therapeutic dose does not cause the “cheese” reaction. The potential of selegiline to modify disease progression in PD was first proposed when it was shown to prevent 1-methyl-4-phenyl-1,2,3,6-tetrahydropyridine (MPTP)-induced parkinsonism in monkeys [4]. *Invitro and invivo* experiments have reported neuroprotective properties of selegiline [4-11]. Selegiline is a derivative of methamphetamine and is metabolized to L-amphetamine-like metabolites which can cause sympathomimetic side effects such as insomnia [12].

### Selegiline as monotherapy or an adjunct to levodopa

Selegiline monotherapy was shown to provide modest symptomatic benefit and delay the need of levodopa therapy in early PD [13-15]. As an adjunct to levodopa therapy, selegiline can reduce motor fluctuations [15].

Deprenyl and Tocopherol Antioxidative Therapy of Parkinsonism (DATATOP) was the largest prospective controlled trial ever done for Selegiline [13]. The DATATOP study was initially designed to evaluate the neuroprotective properties of selegiline and tocopherol. Eight hundred untreated *de novo* PD patients were randomly assigned according to a 2x2 factorial design to one of the four treatment arms: selegiline placebo and alpha-tocopherol placebo; selegiline 10 mg/day and alpha-tocopherol 2000 IU/day; selegiline 10 mg/day; and alpha-tocopherol 2000 IU/day. Unified Parkinson’s Disease Rating Scale (UPDRS) were evaluated at 1 month and 3 months after randomization, then approximately 3 monthly for a planned maximum of 2 years. The primary end point was reached when subjects developed a level of functional disability which required levodopa therapy. There was significant improvement of UPDRS score in the subjects who received selegiline during the 3 months “wash in” period indicating an early symptomatic benefit of selegiline. Selegiline delayed the need of levodopa by approximately 9 months. The Kaplan-Meier analysis showed that taking selegiline significantly reduced the probability of having to start levodopa therapy during the study period (hazard ratio 0.50; 95% confidence interval 0.41 to 0.62, p<0.001). However, after a “wash out” period in subjects who did not reach the end point, there was a significant deterioration of the UPDRS score, indicating a symptomatic effect of selegiline. This symptomatic effect was not factored in during the initial study design. The results of DATATOP are generally considered as being significantly confounded by the symptomatic effects of selegiline.

Further evidence supporting the role of selegiline in the treatment of PD came from another multicentered, randomized, placebo-controlled, double-blinded study, involving 157 patients, who were randomly assigned to receive either selegiline 10 mg/day or placebo [14]. The primary end point was reached when initiation of levodopa therapy became necessary. At 3 months follow up, the selegiline group had significant improvement of UPDRS total score (−1.7±5.4 vs. 1.0±5.3, p<0.01), Visual Analogue Scale (VAS) tremor score (−4.0±18.4 vs. 4.0±16.9, p<0.05) and VAS motor dysfunction score (−3.0±21.3 vs. 6.8±19.6, p<0.05), when compared to the placebo group. The need for levodopa was delayed by 4.1 months with selegiline (p=0.028). In their follow up study up to 7 years involving 141 patients, either selegiline or placebo was restarted in addition to levodopa therapy after an initial 8 weeks “wash out” period [16]. The selegiline group had slower disease deterioration as measured by the UPDRS total score (p=0.003), motor (p=0.002) and ADL (p=0.0002) subscores. Considering both the initial monotherapy and subsequent combination therapy up to 7 years, selegiline did not delay the start on wearing off fluctuations (hazard ratio 0.55; 95% confidence interval: 0.28 to 1.07, p=0.076).

A recent systemic review supported the early symptomatic and long term benefit of selegiline [15]. Selegiline was shown to be beneficial compared to control in motor impairment in 4 randomized control trials (RCTs) involving 986 patients. The weighted mean difference (WMD) for the change in motor UPDRS score was −4.49 (95% confidence interval: -5.52 to −3.46) and WMD in UPDRS ADL score was −2.19 (95% confidence interval: -2.78 to −1.60) at 1 year. Motor fluctuations were significantly reduced with selegiline (6 RCTs involving 1461 patients, odds ratio 0.73; 95% confidence interval: 0.58 to 0.91) at a mean weighted duration of follow up of 3.4 years. There was no significant difference in death or dyskinesia over the control subjects.

### Selegiline in clinical trials for disease-modification in PD

There is no conclusive evidence from clinical trials to prove that selegiline has “disease-modification” effects, even though it was shown to have neuroprotective properties in *invitro and invivo* experimental models [4-11]. Long term clinical trials of selegiline have shown improved motor outcome and reduced levodopa requirement [16-19]. Whether these findings were attributed to the symptomatic benefits or the disease-modification property of selegiline remain debatable. Unlike rasagiline in which delayed-start design trials were carried out in an attempt to separate confounding symptomatic effects from disease-modifying effects, there are none for selegiline (discussed in more detail below).

### Lazabemide

Lazabemide (N-(2-aminoethyl)-5-chloro-2-pyridinecarboxamide) was first tested in clinical trials for treatment of PD in the 1990s. Lazabemide is a more selective inhibitor of MAOB when compared to selegiline. Unlike selegiline, it is not metabolized to L-amphetamine-like metabolites and has a shorter “wash out” period [20]. The shorter “wash out” period of lazabemide was thought to provide a better assessment of the disease-modifying effects, as the change in PD status between baseline and post-treatment after a “wash out” period may not be confounded by any persistent symptomatic effect [21]. The disease-modifying effects of lazabemide was assessed in a randomized controlled trial which involved 321 *de novo* early PD patients [21]. Patients were randomly assigned to receive either placebo or lazabemide 25 to 200 mg/day with a 2 to 4 week “wash out” period. Similar to selegiline, lazabemide was found to have a mild symptomatic effect. The probability of reaching disability sufficient to need initiation of levodopa therapy was significant reduced in lazabemide-treated patients (hazard ratio 0.49; 95% confidence interval 0.32 to 0.77; p=0.001). After the “wash out” period, lazabemide-treated patients had a slower deterioration of PD impairment as evidenced by a lesser decline in total UPDRS score when compared to placebo (− 6.1±8.4 vs. – 8.1±8.5; p=0.06), indicating a possible disease-modifying effect. However, its manufacturer decided to halt further development of lazabemide in 1999 after reporting liver toxicity [22].

### Rasagiline

Rasagiline (N-propargyl-1-(R)-aminoindan) is a second generation propargylamine-based selective, irreversible MAOB inhibitor. It was reported to have potent anti-apoptotic effects independent of MAO inhibition in *invitro* and *invivo* experimental parkinsonian models [23-25]. It can cross the blood–brain-barrier readily [26]. Similar to selegiline, rasagiline lacks dietary tyramine sympathomimetic potentiation at its MAOB inhibitory dosage [23,27], and hence, lacks the undesirable pressor effects of the “cheese” reaction associated with MAOA inhibition. Unlike selegiline, rasagiline is not metabolized to L-amphetamine-like metabolites which may cause appetite suppression and insomnia [24].

### Rasagiline as an adjunct therapy to levodopa for motor fluctuations in PD

Rasagiline was tested as an adjunct therapy to levodopa in alleviating parkinsonian motor fluctuations in two separate multicentered, double-blind, parallel-group clinical trials: a) PRESTO (Parkinson’s Rasagiline: Efficacy and Safety in the Treatment of “Off”) [28] and b) LARGO (Lasting effect in Adjunct therapy with Rasagiline Given Once daily) studies [29].

In the PRESTO study, 472 PD patients with at least 2½ hours of daily “off” time were randomized to receive rasagiline (in either 1 mg/day or 0.5 mg/day) or placebo, as an adjunct therapy to levodopa over 26 weeks [28]. In its primary outcome measure, the mean adjusted total daily “off” time as measured by patients’ home diaries had a modest but statistically significant reduction from baseline by 1.85 hours (29%) in patients treated with 1 mg/day dose, by 1.41 hours (23%) with 0.5 mg/day dose, and 0.91 hours (15%) with placebo. Patients had 0.94 hours and 0.49 hours less “off” time on 1 mg/day and 0.5 mg/day doses respectively, compared to placebo. The changes on activities of daily living (ADL) and quality of life were less consistent. The Clinical Global Impression (CGI) and the UPDRS ADL scores during “off” time showed improvement as secondary end points with both doses of rasagiline, but not with PDQUALIF (PD Quality of Life) summary score.

In the LARGO study, 687 PD patients with at least 1 hour of “off” time during awake hours as measured by 24-hour patients’ home diaries, were randomized to receive either rasagiline (1 mg/day), entacapone (200 mg with each dose of levodopa) or placebo, as an adjunct therapy to levodopa for 18 weeks [29]. In its primary outcome measure, patients who had received rasagiline had a statistically significant reduction of mean daily “off” time by 1.18 hours; those on entacapone by 1.2 hours (both about 20%), and placebo by 0.4 hours from baseline. The mean daily “on” time without troublesome dyskinesia had a modest but statistically significant increase of 0.85 hours in those patients who received rasagiline compared with 0.03 hours who received placebo; the improvement was similar in magnitude to entacapone. The CGI and UPDRS ADL scores during “off” time significantly improved in those patients taking rasagiline compared to placebo, comparable to entacapone.

In both the PRESTO and LARGO trials, rasagiline was well-tolerated. The adverse events which included nausea, anorexia and postural hypotension were mild. Rasagiline did not have any significant effects on the pulse or blood pressure.

### Rasagiline in clinical trials for disease-modification in PD

Apart from its effects on the motor symptoms of PD, rasagiline was tested for its neuroprotective effects in two prospective, double-blind, placebo-controlled, parallel-group, randomized clinical studies on patients with early PD: a) TEMPO (TVP-1012 in Early Monotherapy for PD Outpatients) [30,31], and b) ADAGIO (Attenuation of Disease Progression with Azilect Given Once-daily) studies [32]. Patients with early stages of untreated PD were chosen because of the concern that the pathogenic processes and neuronal cell death would have been too far advanced in the late stages of PD for disease-modification to make any clinically meaningful impact. Another major concern raised after the conclusion of the DATATOP study and the subsequent studies on selegiline, was the confounding effects of symptomatic improvement on the assessment of disease-modifying benefits of the drug [13].

Both TEMPO and ADAGIO studies used a delayed-start design in an attempt to separate confounding symptomatic from disease-modifying effects [30-32]. The design essentially divided the trial period into two phases. The treatment groups would be randomized in the first phase to either placebo or rasagiline treatment arms. The first phase would have to be sufficiently long to allow any disease-modifying effects of rasagiline more opportunity to manifest. This initial phase would be followed by a second phase where all the groups would receive rasagiline but the initial randomization was kept blinded. As all the treatment arms received rasagiline during the second phase of the study, any confounding symptomatic effects between the different arms at the end of the study would be negated. Hence, any differences in outcome would presumably be due to disease-modifying effects of rasagiline from the delayed start of rasagiline during the first phase of the study.

In the TEMPO study, 404 early PD patients who did not require dopaminergic therapy were randomized to three parallel arms: 1 mg/day or 2 mg/day for 12 months, or placebo for the first 6 months followed by rasagiline 2 mg/day for another 6 months [30,31]. In its analysis based on the primary outcome measure over the first 6 month trial period, the total UPDRS score (adjusted for effect size) worsened significantly less compared to placebo from baseline; by 4.2 points less than placebo (95% confidence interval: 5.66 to 2.73 points, p<0.001) in patients who received rasagiline 1 mg/day, and by 3.56 points less than placebo (95% confidence interval: 5.04 to 2.08 points, p<0.001) in patients who received 2 mg/day dose [30]. The improvement observed was similar between the two doses of rasagiline over this initial 6 month period. Although there are no direct comparisons as yet, the symptomatic effects of rasagiline monotherapy in early PD appear to be more modest than dopamine agonists. In a later analysis performed in the same study, patients who received rasagiline 1 mg/day over 12 months had less worsening of mean adjusted total UPDRS score of 1.82 points (95% confidence interval: 3.64 to 0.01 points, p=0.05) compared with the delayed start rasagiline 2 mg/day [31]. Patients who received rasagiline 2 mg/day over 12 months had even less worsening of mean adjusted total UPDRS score of 2.29 points (95% confidence interval: 4.11 to 0.48 points, p=0.01) compared with the delayed start rasagiline 2 mg/day. The study concluded that rasagiline at either 1 mg/day or 2 mg/day had less functional decline compared with the delayed start group. These studies raised the possibility that early-start rasagiline appeared to have enduring benefits over delayed-start, but it also raised concerns [33]. In an open-labeled extension the TEMPO study, 306 patients who continued on rasagiline were followed up to 6½ years (mean±SD: 3.6±2.1 years) [34]. The adjusted mean difference in change from baseline in total UPDRS scores was 2.5 points (16%) in favor of early-start compared to delayed-start. This extension study also reported that although the interaction between treatment and time was significant, there was significantly less worsening in total UPDRS scores in the early-start compared to the delayed-start group at all seven half-yearly follow up time points.

The ADAGIO study which was larger and longer, but with a similar delayed-start design on rasagiline soon followed [35]. It involved 1,176 patients with early, untreated PD randomized in four parallel arms: rasagiline at either 1 mg/day or 2 mg/day for a total of 72 weeks (early-start group), and placebo for 36 weeks followed rasagiline at either 1 mg/day or 2 mg/day for another 36 weeks (delayed-start group). In order for the study to indicate significant disease-modification, the early-start group had to meet each of three hierarchical ends points of the primary analysis based on the total UPDRS score: a) superiority to placebo in the rate of change of the UPDRS score between weeks 12 and 36 during the first phase of the study, b) superiority to delayed-start treatment in the change of the score from baseline to week 72, and c) non-inferiority to delayed-start treatment in the rate of change of the score between weeks 48 and 72 during the second phase of the study. The results showed that rasagiline 1 mg/day dose achieved all three hierarchical primary endpoints based on disease progression: a) a slower mean rate of worsening in total UPDRS score between weeks 12 and 36 compared to placebo (0.09 points/week in early-start treatment versus 0.14 points/week in placebo group), b) less worsening of mean total UPDRS score from baseline to week 72 in early-start (2.82 points) compared delayed-start (4.52 points) treatment, c) non-inferiority of the early-start treatment in the mean rate of change of the total UPDRS score between weeks 48 and 72 in early-start (0.085 points/week) compared with delayed-start (0.085 points/week) treatment. However, the 2 mg/day dose failed to achieve all its primary endpoints because the change of mean total UPDRS score from baseline to week 72 in the early-start (3.47 points) was not significantly different from the delay-start treatment (3.11 points). However, the early-start treatment group taking rasagiline 2 mg/day dose had less mean rate of worsening (0.07 points/week) in total UPDRS score compared with placebo (0.14 points/week) between weeks 12 to 36. Furthermore, at the 2 mg/day dose, the mean rate of change in total UPDRS between weeks 48 to 72 in the early-start (0.094 points/week) was non-inferior to delayed-start treatment (0.065 points/week). Nevertheless, the result of the 2 mg/day dose was considered to be negative because the design was such that all three hierarchical primary endpoints had to be met for each separate dose. The authors concluded that early-start treatment rasagiline at 1 mg/day provided benefits consistent with possible disease-modifying effects even though it did not met its endpoints at 2 mg/day.

The ADAGIO study results raised some debate and concerns, in particular, the divergent and paradoxical outcome between 1 mg/day and 2 mg/day doses [36,37]. It is unknown how PD progresses and whether it does so linearly. It was also unclear whether confounding symptomatic effects of rasagiline were significantly negated. The first hierarchical endpoint of superiority to placebo in the rate of change of the total UPDRS score between weeks 12 and 36 relied on the assumptions that, a) the rate of change of this score was linear during this period; and b) the symptomatic effects of rasagiline were fully established by week 12 [35]. This linearity from weeks 12 to 36 and whether the confounding symptomatic effects were fully established before the assessments on the first endpoint, have been disputed [36], and questions raised on whether the rasagiline 1 mg/day dose had met the pre-specified criteria. The authors of the ADAGIO trial argued that there are many pharmacological examples where dose-related efficacy may not occur but it was thought that the doubling of the rasagiline dose was unlikely to have a U or J curve effect [38,39]. The authors of the ADAGIO trial also noted that their *posthoc* analysis of the subgroup of patients with the highest total UPDRS score at baseline (i.e., the most severely affected quartile) had met all its primary endpoints with their early-start rasagiline 2 mg/day dose [35]. They postulated that the results could be positive if the rasagiline 2 mg/day dose was tested on more severely affected patients at baseline [39]. However, this could mean a higher drop-out rate especially in the placebo/active treatment group during the first phase of the study which could confound the results. Questions remain that if the trial was extended further in a blinded-manner during the second phase, whether the difference in outcome between the delayed-start and early-start treatment groups would diverge or converge.

### Symptomatic benefits of MAOB inhibitors in early PD when compared with other medications for PD

MAOB inhibitors provide a modest symptomatic benefit in the treatment of early PD. The change in mean total UPDRS score after 3 months of selegiline 10 mg/day was −1.6 points in the DATATOP study [13], while the change in mean score after 6 months of rasagiline 1 mg/day was +0.1 points in the TEMPO study [30]. The deterioration in the UPDRS scores for the treatment groups in both studies was significantly slower when compared to placebo, indicating a symptomatic benefit of MAOB inhibitors. Although there are no direct comparisons as yet, the symptomatic benefits of MAOB inhibitors were weaker when compared to dopamine agonist or levodopa. The change of mean motor UPDRS score was −4.5 points for ropinirole, -3.4 points for pramipexole and −7.3 points for levodopa after 6 months of treatment [40,41].

### Obstacles to clinical trials in disease-modification for PD

There are inherent problems with PD which may form insurmountable barriers to disease-modifying strategies in clinical trials. There are no consistently reliable biological or neuroimaging markers in PD. As yet, the diagnosis of PD is still made clinically. There are no similar markers to correlate with disease progression. Its definition, progression and primary outcome measures are still determined by its clinical features in many trials. Furthermore, its clinical features are heterogeneous, from tremor-predominant to akinetic-rigid forms, younger onset patients with more dystonic features, and older onset patients with more dementia [42,43]. Its progression also varies between different patients, and may differ within the same patient at different points in time [44-46]. The UPDRS is inadequate to address the many complexities associated with PD symptomatology. This score is focused primarily on dopaminergic responsive symptoms, and is poorly adapted to help define and monitor non-motor features which are more disabling, and less responsive to treatment. Various efforts to improve the UPDRS and new diagnostic criteria for non-motor features are being established in an effort to address these concerns [47-49]. This task is made more difficult considering that PD patients can have fluctuations in motor and non-motor clinical features even within the same day. Perhaps the most difficult issue is that the cause or causes of PD are unknown. PD may have heterogeneous etiologies and probably multiple pathogenic pathways. Clinical trials using single or even double agents designed to modify the course of a homogeneous disorder may well never achieve its aim. Conducting clinical trials is expensive, especially when combinations of therapies are tested for extended periods. There is still no clear consensus on what constitutes a disease-modifying therapy as there are many caveats to its definition.

### Potential benefits of early treatment in normalizing compensatory mechanisms in PD

**Figure 2:**
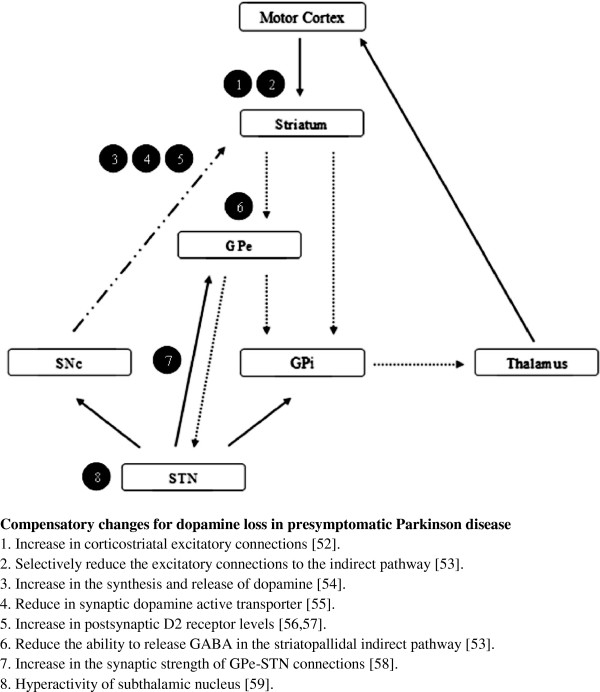
**Compensatory changes for dopamine loss in pre-symptomatic Parkinson's disease.** Abbreviations: GPe, globus pallidus pars externa; GPi, globus pallidus pars interna; STN, subthalamic nucleus; SNc, substantia nigra pars compacta; GABA, gamma-aminobutyric acid. Solid arrows: excitatory glutamatergic connections; Dot arrows: inhibitory GABAergic connections; Dash dot arrow: dopaminergic connections.

Disease-modifying therapy should intuitively have the most impact at the earliest stages of the disease when there are still functional neuronal networks to be preserved. Indirect evidence from the ELLDOPA (Earlier versus Later Levodopa Therapy in Parkinson Disease) [50], and TEMPO trials have shown that symptomatic therapies started early in the disease may help to reduce the severity in the latter stages of the disease compared with a later start [34]. Schapira and Obeso had proposed that compensatory changes in the basal ganglia circuitries and thalamo-cortical projections occur in the earlier stages of PD to maintain its physiological motor function in response to a gradual deficit in striatal dopamine associated with degeneration of the nigrostriatal pathway [51]. Compensatory changes can occur at the nigrostriatal pathway and the basal ganglia circuit [52-59] (Figure 2). They further proposed when the dopaminergic deficit in PD surpasses the threshold of basal ganglia compensatory mechanisms, motor symptoms develop associated with hyperactivity in globus pallidum externa-subthalamic nucleus-globus pallidum interna pathway and loss of modulation for normal basal ganglia output. A major feature of this basal ganglia imbalance is potentially deleterious glutamatergic hyperactivity arising from the subthalamic nucleus, pedunculopontine nucleus, intra-laminar thalamic nucleus, and corticostriatal projection, exacerbating excitotoxicity and other pathological mechanisms. Earlier restoration of the dopaminergic deficit with early symptomatic treatment may restore this imbalance to a more normal state, and reduce deleterious pathological mechanisms which can exacerbate the progression of the disease. It is unclear whether early symptomatic treatment is associated with long term disease-modifying effects. Their hypothesis indicates that symptomatic and disease-modifying effects in clinical trials are not mutually exclusive [60].

## Conclusions

Tables 1 and 2 summarize the pharmacology properties, therapeutic benefits and major trials for selegiline and rasagiline. There is little to choose between selegiline and rasagiline, although the latter does not have amphetamine-like side effects which may cause appetite suppression and insomnia. Unlike the laboratory evidence, there is currently no conclusive proof from existing clinical trials that MAOB inhibitors have disease-modification effects on the natural history of PD. Questions remain whether the results of the disease-modification trials reflect genuine disease-modifying effects, or confounded by the limitations of the clinical trials and the measures of efficacy. However, the potential benefits of MAOB inhibitors with little or well-tolerated side effects may be useful in some patients with PD, especially in younger patients with milder symptoms as monotherapy or as an adjunct to levodopa.

**Table 1:** Pharmacology properties and therapeutic benefits of MAOB inhibitors in clinical use for Parkinson's disease

	**Selegiline**	**Rasagiline**
Recommended dosage	10 mg daily	1 mg daily
Bioavailability	Unknown [61]	36% [62]
Half-life	10 hours [61]	0.6 – 2 hours [62]
Metabolite	L-amphetamine like metabolites [61]	Aminoindan [62]
Symptomatic monotherapy	Efficacious [15]	Efficacious [30,35]
Adjunct to levodopa and treatment to motor complications	Likely efficacious [15]	Efficacious [28,29]
	- Levodopa sparing effect	- Levodopa sparing effect
	- Reduction in motor fluctuation	- Reduction in motor fluctuation
Disease-modification	Insufficient evidence	Insufficient evidence

**Table 2:** Major trials for selegiline and rasagiline

**Selegiline**		**Rasagiline**	
DATATOP 1993 [13]		TEMPO 2004 [31]	
Study design	RCT selegiline vs. placebo	Study design	Randomized, delayed start trial rasagiline for 1 year vs. 6 months placebo then 6 months rasagiline
Participants	800 patients	Participants	404 patients
Follow up period	2 years	Follow up period	1 year
End point	Functional disability requiring levodopa	End point	Change in UPDRS score
Major finding	Need for levodopa delayed by 9 months in the selegiline group	Major finding	Slower disease deterioration in the early start rasagiline group
Conclusion	Symptomatic benefit	Conclusion	Symptomatic benefit
			Possible disease-modifying effect
SELEDO 1999 [19]		PRESTO 2005 [28]	
Study design	RCT selegiline vs. placebo, as adjunct to levodopa	Study design	RCT rasagiline vs. placebo, as adjunct to levodopa
Participants	116 patients	Participants	472 patients with daily off time
Follow up period	5 years	Follow up period	26 weeks
End point	Increase in >50% of initial levodopa dose	End point	Total daily off time
Major finding	Primary end point delayed in the selegiline group	Major finding	Less off time in the rasagiline group
Conclusion	Symptomatic benefit as adjunct to levodopa	Conclusion	Symptomatic benefit as adjunct to levodopa
Larsen 1999 [17]		LARGO 2005 [29]	
Study design	RCT selegiline vs. placebo, as adjunct to madopar. 1 month wash out of selegiline at the end of study period	Study design	RCT rasagiline vs. entacapone vs. placebo, as adjunct to levodopa
Participants	163 patients	Participants	687 patients with daily off time
Follow up period	5 years	Follow up period	18 weeks
End point	Levodopa requirement and deterioration of UPDRS score	End point	Total daily off time
Major finding	Lower levodopa requirement and UPDRS score in the selegiline group	Major finding	Less off time in the rasagiline group
Conclusion	Symptomatic benefit as adjunct to madopar	Conclusion	Symptomatic benefit as adjunct to levodopa
	Possible disease-modifying effect		
Palhagen 2006 [16]		ADAGIO 2009 [35]	
Study design	RCT selegiline vs. placebo, as adjunct to levodopa	Study design	Randomized, delayed start trial rasagiline for 72 weeks vs. 36 weeks placebo then 36 weeks rasagiline
Participants	140 patients	Participants	1176 patients
Follow up period	7 years	Follow up period	72 weeks
End point	Deterioration of UPDRS score	End point	Three hierarchical end points to indicate significant disease-modification
Major finding	Slower disease deterioration in the selegiline group	Major finding	Rasagiline 1 mg/day achieved all three hierarchical end points, but not in 2 mg/day dose
Conclusion	Symptomatic benefit as adjunct to levodopa	Conclusion	Possible disease-modifying effect

## Competing interests

Both authors have no conflict of interest. The corresponding author is a subsection editor of Translation Neurodegeneration.

## Authors’ contributions

KC and SL drafted the manuscript. Both authors read and approved the final manuscript.
